# Supplementary material for: High-Level Production of a Thermostable Mutant of Yarrowia lipolytica Lipase 2 in Pichia pastoris
Source: Int J Mol Sci. 2019 Dec 31;21(1):279. doi: 10.3390/ijms21010279 (PMC6982173; doi:10.3390/ijms21010279)
Supplement: Supplementary file 1 [file ijms-21-00279-s001.pdf]

Supplementary Data

High-level Production of a Thermostable Mutant of *Yarrowia lipolytica* Lipase 2 in *Pichia pastoris*

Qinghua Zhou†, Zhixin Su†, Liangcheng Jiao, Yao Wang, Kaixin Yang, Wenjuan Li, Yunjun Yan *

Key Laboratory of Molecular Biophysics, the Ministry of Education; College of Life Science and Technology, Huazhong University of Science and Technology, Wuhan 430074, P. R. China; qinghuazhou1@126.com (Q.Z.); xuzhixin5512@163.com (Z.S.); jiaoliangcheng@gmail.com (L.J.); wangyao_1962@163.com (Y.W.); ykxllq@163.com (K.Y.); 18238636670@139.com(W.L.)

* Correspondence: yanyunjun@hust.edu.cn; Tel.: +86-27-87792213

† These authors contributed equally to this work.

Table S1 The optimized culture parameters for protein expression in *P. pastoris*.

**Proteins**	**Temperature (°C)**	**Initial pH**	**Methanol concentration (*v/v*)**	**Culture medium volume (mL)**	**Inoculation density (*v/v*)**	**Incubation time (h)**	**References**
YlLip2 mutant	22	7.0	1%	30	3%	144	This study
Wild-type YlLip2	25	6.5	1%	-	-	-	[1]
Rhizopus oryzae lipase	27	7.0	1.2%	20	4%	120	[2]
Pycnoporus sanguineus laccase	30	6.5	0.5%	-	-	168	[3]
Human μ-opioid receptor	20	-	0.5%	-	-	-	[4]
G-protein-coupled receptors	20	7.0	0.5%	-	-	-	[5]

Table S2 Plasmids and strains used in this study.

Plasmids/strains	Description	Source or reference
**Plasmids**		
pPICZαA (△ *Sal*I)	stored in our laboratory, deleted the *Sal*I site	
pAOα-ROL	pAO815 carrying *rol* gene	[2]
pPICZαA-lip2	pPICZαA derivative, carrying *lip2* gene	This study
pAOα-lip2	pPICZαA-lip2 derivative, carrying *lip2* gene	This study
pAOα-2lip2	pAOα-lip2 derivative, carrying 2 copies of *lip2* gene	This study
pAOα-3lip2	pAOα-2lip2 derivative, carrying 3 copies of *lip2* gene	This study
pAOα-4lip2	pAOα-3lip2 derivative, carrying 3 copies of *lip2* gene	This study
pPICZA-hac1	pPICZA carrying *hac1* gene	[2]
pPIC3.5k-hac1	pPIC3.5K carrying *hac1* gene	This study
pPICZA-vgb	pPICZA carrying *vgb* gene	[6]
pMD19-GAP	pMD19T carrying GAP gene	[2]
**Strains**		
E. coli Top10	*E. coli* host strain	Invitrogen
GS115	*P. pastoris* host strain	Invitrogen
GS115/pAOα-lip2	GS115 harboring pAOα-lip2	This study
GS115/pAOα-2lip2	GS115 harboring pAOα-2lip2	This study
GS115/pAOα-3lip2	GS115 harboring pAOα-3lip2	This study
GS115/pAOα-4lip2	GS115 harboring pAOα-4lip2	This study
GS115/2lip2-3.5Khac1	GS115 harboring pAOα-2lip2 and pPIC3.5k-hac1	This study
GS115/2lip2-ZAvgb	GS115 harboring pAOα-2lip2 and pPICZA-vgb	This study
GS115/2lip2-hac1-vgb	GS115 harboring pAOα-2lip2, pPIC3.5k-hac1 and pPICZA-vgb	This study

Table S3 Primers used in this study.

Primers	Sequence (5′-3′)	Annotation
lip2-F	GGAATTCGTGTACACCTCTACCGAGACC	PCR for *lip2* gene, *Eco*RI site
lip2-R	ATTTGCGGCCGCTTAGATACCACAGACACCCTC	PCR for *lip2* gene, *Not*I site
Bgl-F1	ACTAAGATTTTCAAGCCCTTCAACTGTG	PCR for *lip2* gene
BglR1	CACAGTTGAAGGCTTGAAAATCTTAGT	PCR for *lip2* gene
Bgl-F2	A GCAAATCTACCTTGTTATTCGAG	PCR for *lip2* gene
Bgl-R2	CTCGAATAACAAGGTAGATTTGCT	PCR for *lip2* gene
lip2-F1	GGTTACCAGCACGCCTCTGGTG	PCR for *lip2* gene
lip2-R1	CACCAGAGGCGTGCTGGTAACC	PCR for *lip2* gene
his4-F	ATGACATTTCCCTTGCTACC	PCR for *his4* gene
his4-R	TTAAATAAGTCCCAGTTTCTCC	PCR for *his4* gene
QGAP-F	CGGTGTTTTCACCACTTTGGA	qPCR for GAP gene
QGAP-R	CAACGAACATTGGAGCATCCT	qPCR for GAP gene
Qlip2-F	CTCTGGTGAGGTCTTTATTGACTGG	qPCR for *lip2* gene
Qlip2-R	CCGGCAGAGCACTGTTTATTG	qPCR for *lip2* gene
hac1-F	CGGAATTCATGCCCGTAGATTC	PCR for *hac1* gene, *Eco*RI site
hac1-R	ATTTGCGGCCGCCTATTCCTGGAAG	PCR for *hac1* gene, *Not*I site
Qhac1-F	CGCTGAATATGACGACGAAGA	qPCR for *hac1* gene
Qhac1-R	TCTCCTGCTTGATAGATGTGCTC	qPCR for *hac1* gene
Qvgb-F	CCTGCGGTCAAAAAAATTGC	qPCR for *vgb* gene
Qvgb-R	TCTTTAATCGCACCCAACAATTC	qPCR for *vgb* gene

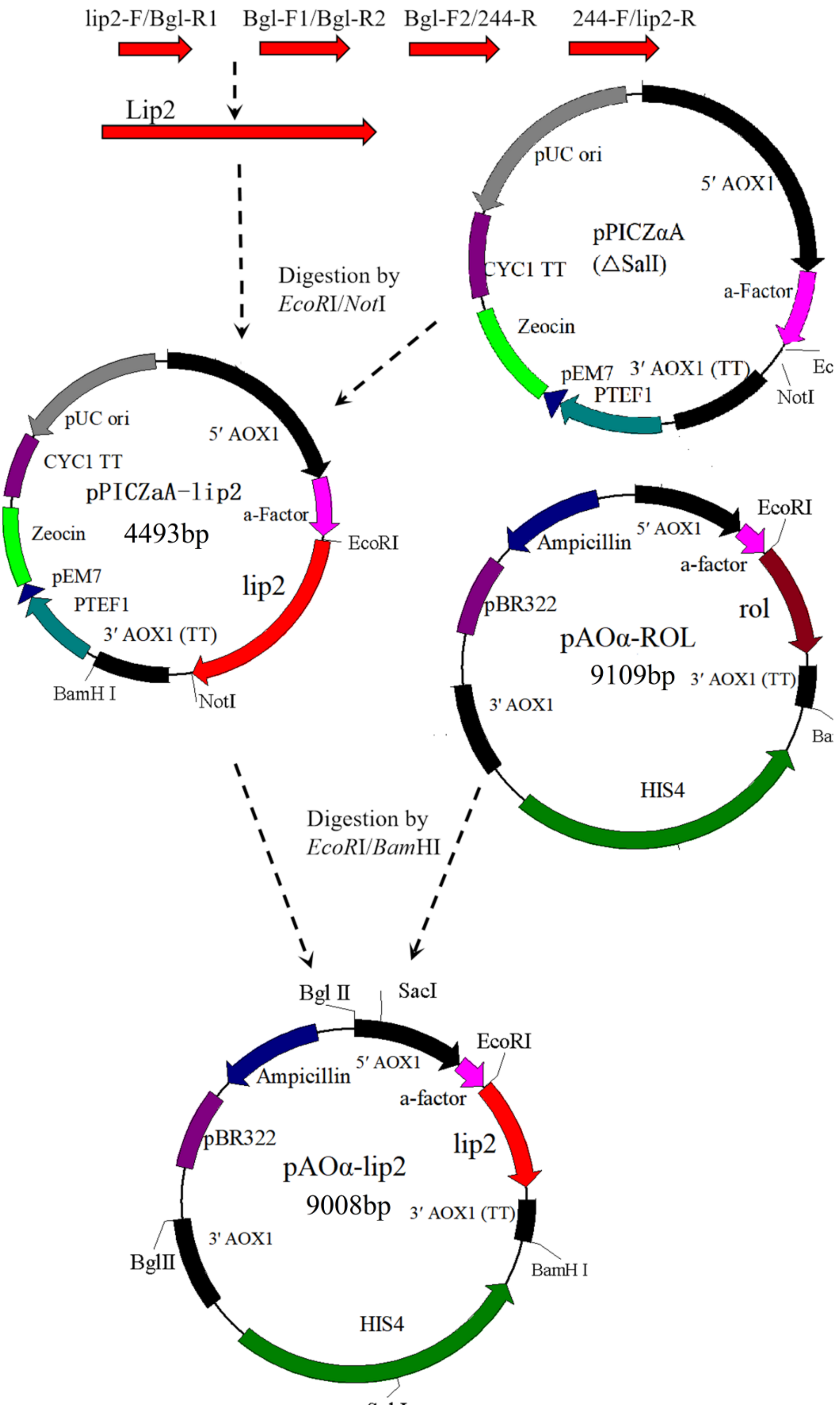

Figure S1. Process diagram for constructing the pAOα-lip2 vector.

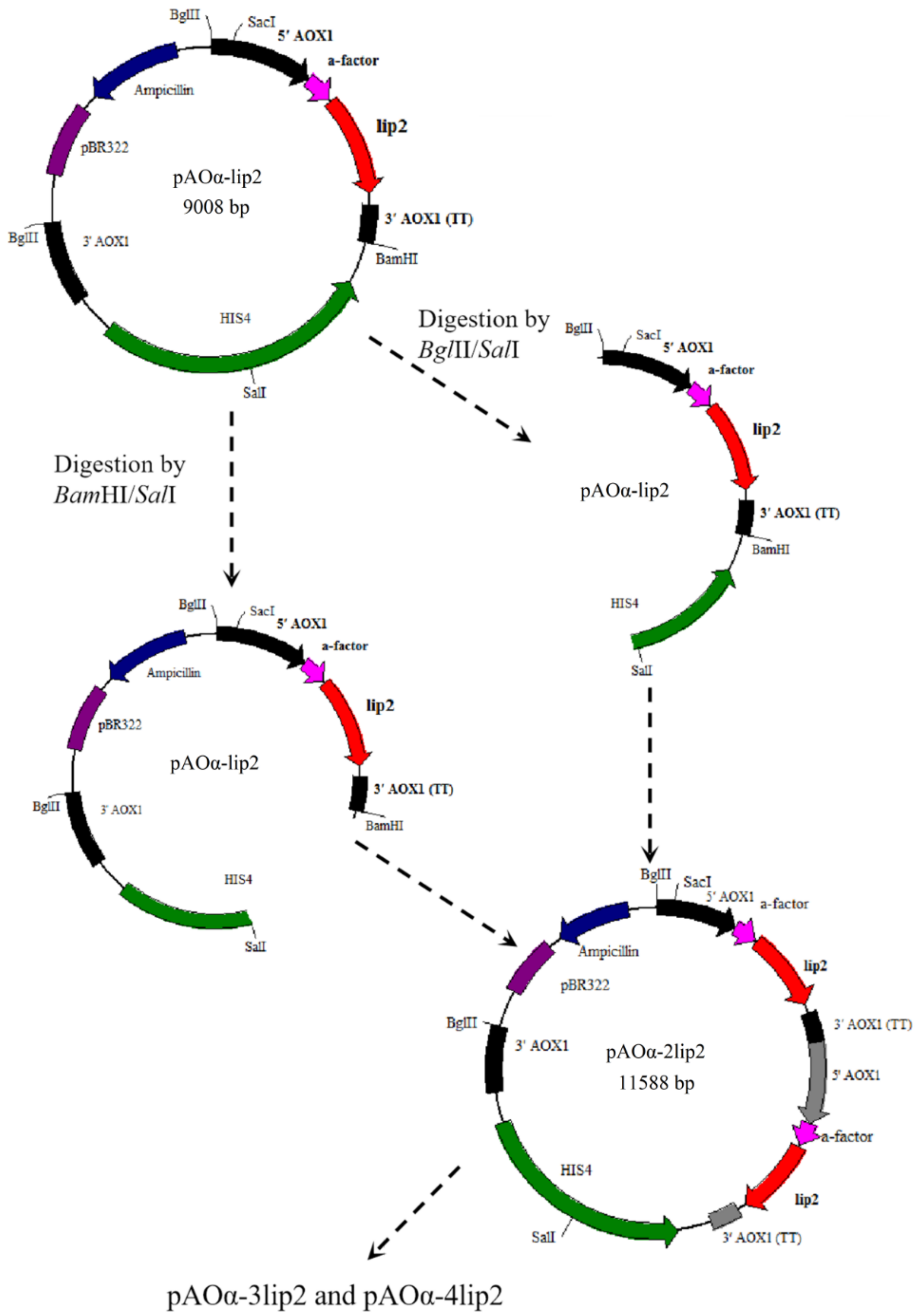

Figure S2. Process diagram for constructing expression vectors pAO*α*-nlip2 (n=2~4).

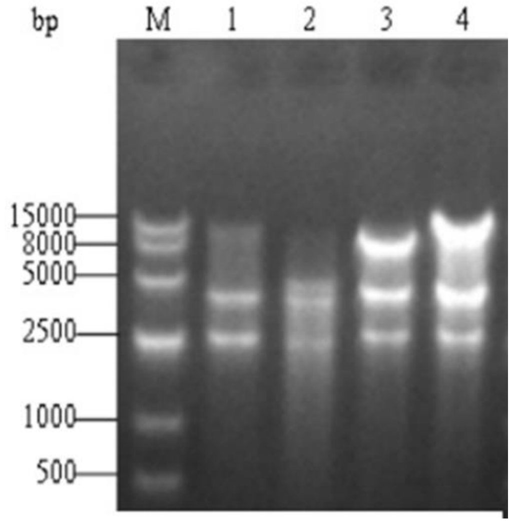

Figure S3. The vectors pAOα-nlip2 (n=1~4) were confirmed by digestion with *Bgl*II and *Bam*HI. Lane M, Marker; lanes 1-4, pAOα-nlip2 (n=1~4).

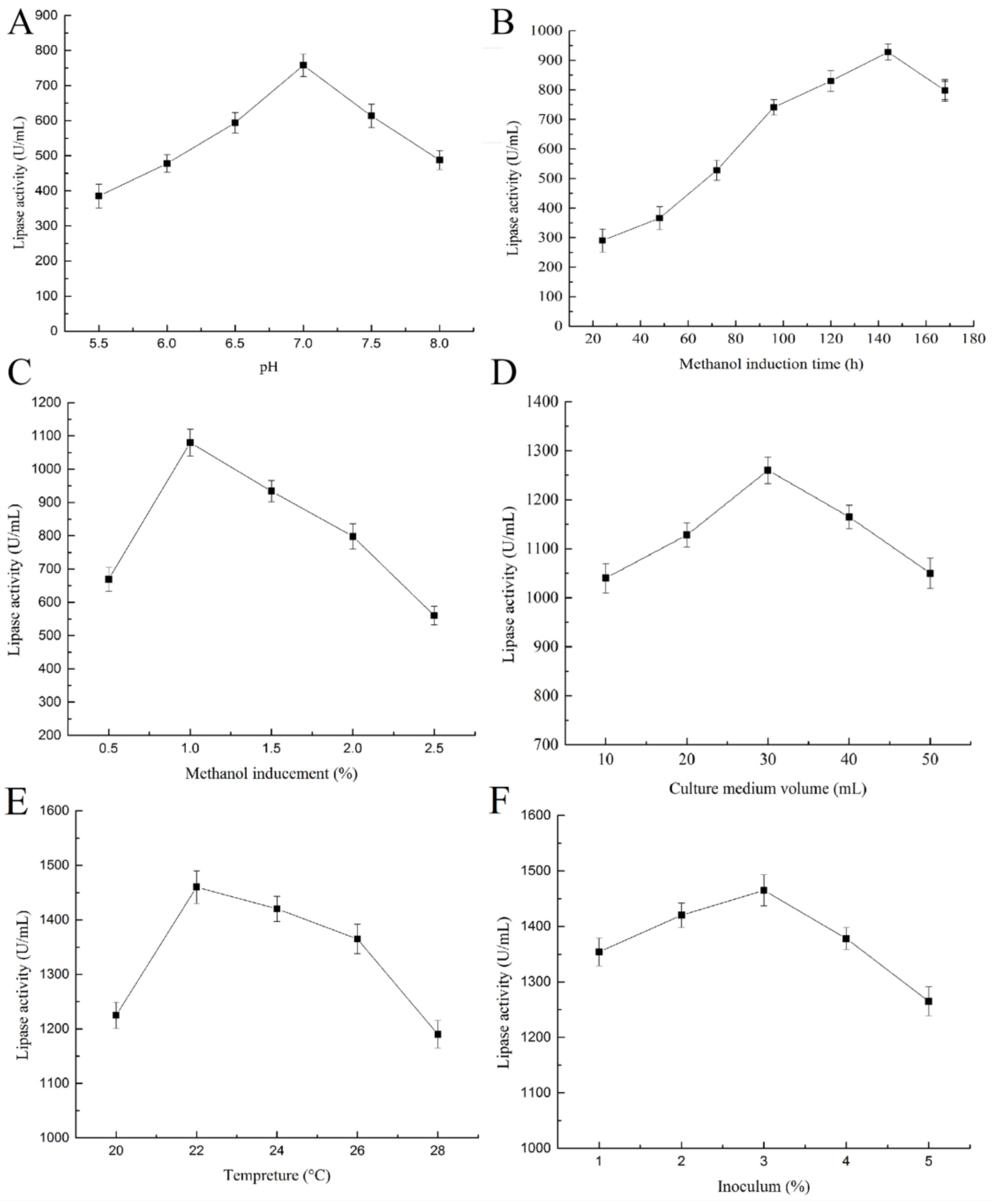

Figure S4. Shaking flask culture optimization for YlLip2 production in *P. pastoris*. (A) Initial pH; (B) Incubation time; (C) Methanol concentrations; (D) Culture-medium volumes; (E) Induction temperatures; (F) Inoculation density. Data are the mean ± standard deviation of triplicate experiments.

1 VYTSTETSHI DQESYNFFEK YARLANIGYC VGPGTKIFKP FNCGLQCAHF

51 PNVELIEEFH DPRLIFDVSG YLAVDHASKQ IYLVIRGTHS LEDVITDIRI

101 MQAPLTNFDL AANISSTATC DDCLVHNGFI QSYNNTYNQI GPKLDSVIEQ

151 YPDYQIAVTG HSLGGAAALL FGINLKVNGH DPLVVTLGQP IVGNAGFANW

201 VDKLFFGQEN PDVSKVSKDR KLYRITHRGD IVPQVPFWDG YQHASGEVFI

251 DWPLIHPPLS NVVMCQGQSN KQCSAGNTLL QQVNVIGNHL QYFVTEGVCG

301 I

Figure S5. Mass spectrometry results for YlLip2 in *P. pastoris*, matched peptides were marked in red.

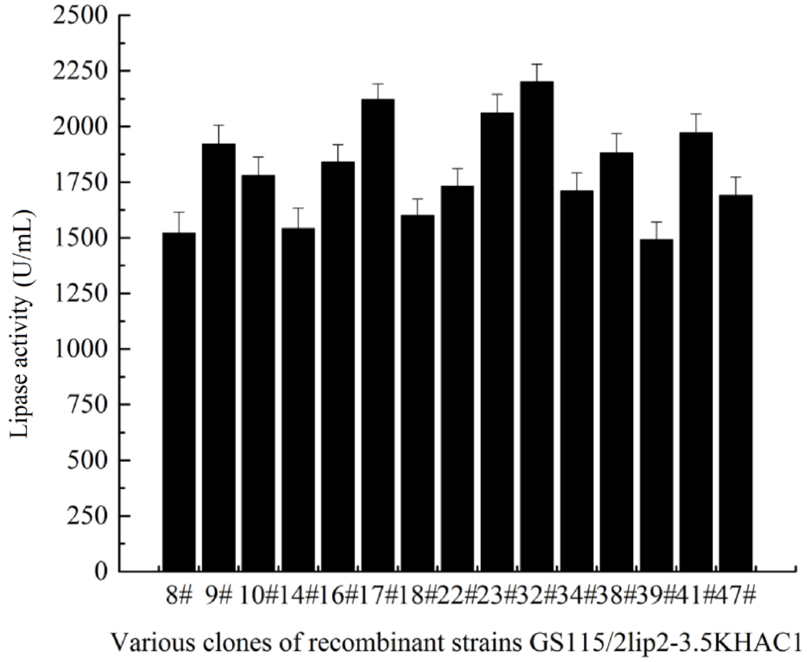

Figure S6. Lipase activity of different colonies from GS115/2lip2-3.5Khac1. Data are the mean ± standard deviation of triplicate experiments.

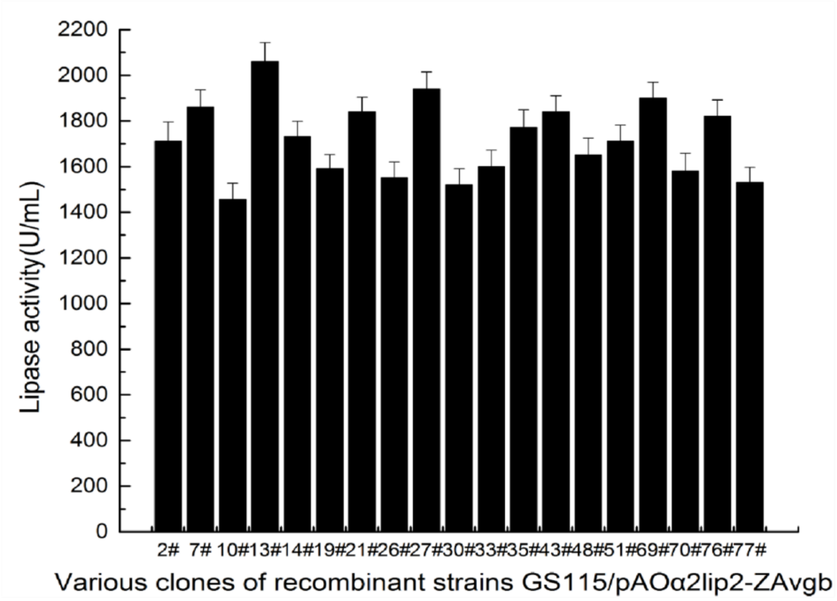

Figure S7. Lipase activity of different colonies from GS115/2lip2-ZAvgb. Data are the mean ± standard deviation of triplicate experiments.

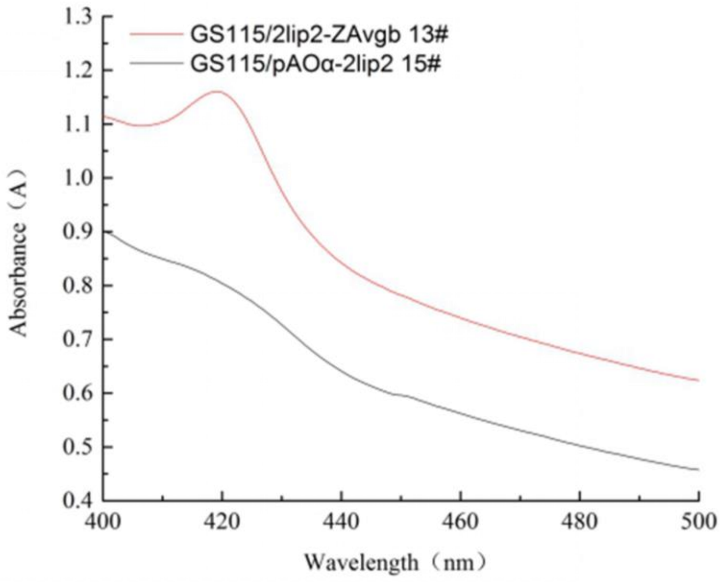

Figure S8. CO-difference spectrophotometric analysis for VHb⁺ strain (GS115/2lip2-ZAvgb 13#) and VHb⁻ strain (GS115/pAOα-2lip2 15#).

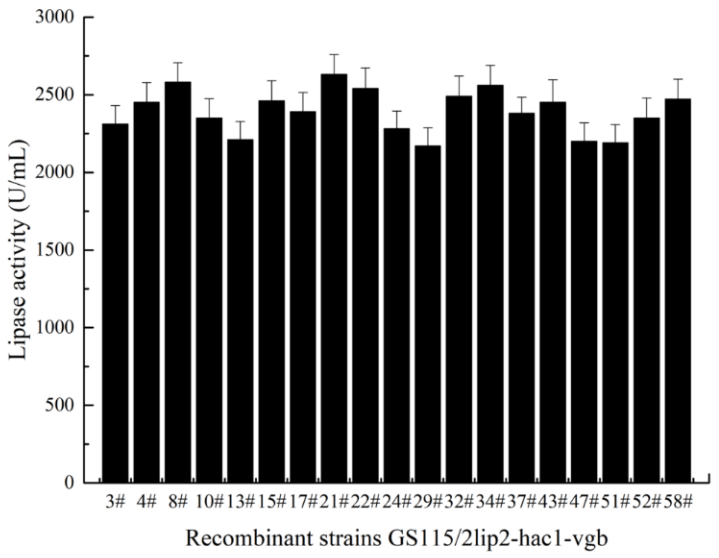

Figure S9. Lipase activity of different colonies from GS115/2lip2-hac1-vgb. Data are the mean ± standard deviation of triplicate experiments.

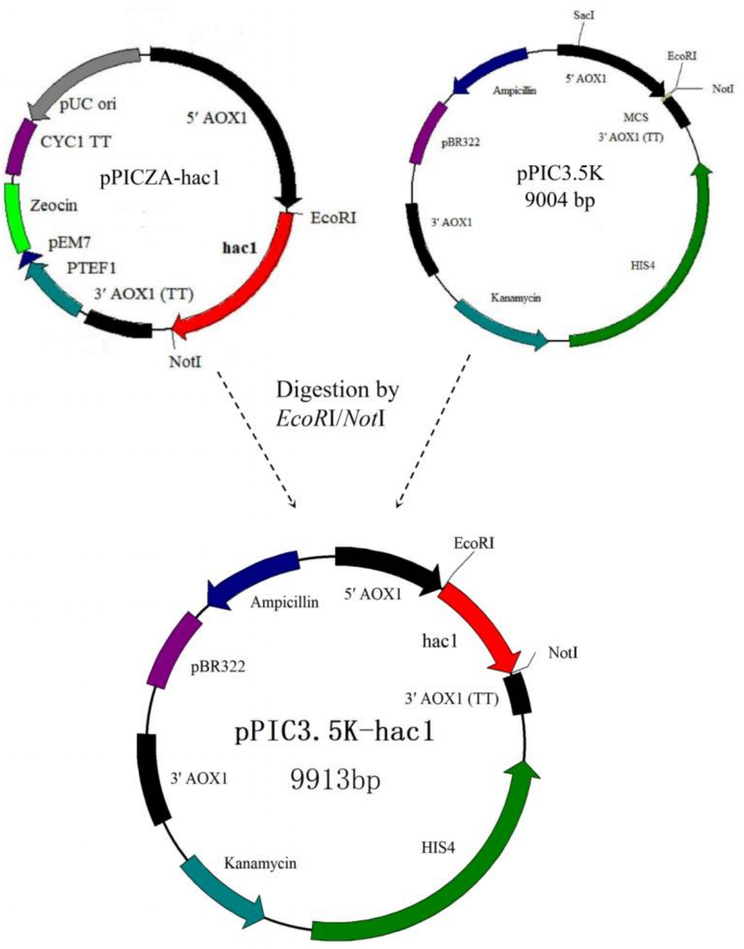

Figure S10. Process diagram for constructing the pPIC3.5k-hac1 vector.

References

1. Wang, X.; Shen, X.; Zhao, H.; Sun, Y.; Liu, T.; Liu, Y.; Xu, L.; Yan, Y. Combined strategies for the improvement of heterologous expression of a His-tagged *Yarrowia lipolytica* lipase Lip2 in *Pichia pastoris*. *Afr. J. Biotechnol*. **2011**, 10(80), 18503-18512.
2. Jiao, L.; Zhou, Q.; Su, Z.; Xu, L.; Yan, Y. High-level extracellular production of *Rhizopus oryzae* lipase in *Pichia pastoris* via a strategy combining optimization of gene-copy number with co-expression of ERAD-related proteins. *Protein Expr. Purif.* **2018**, 147, 1-12. https://doi.org/10.1016/j.pep.2018.02.005.
3. Lu, L.; Zhao, M.; Liang, S.; Zhao, L.; Li, D.; Zhang, B. Production and synthetic dyes decolourization capacity of a recombinant laccase from *Pichia pastoris*. *J. Appl. Microbiol*. **2009**, 107(4), 1149-1156. https://doi.org/10.1111/j.1365-2672.2009.04291.x.
4. Sarramegna, V.; Demange, P.; Milon, A.; Talmont, F. Optimizing functional versus total expression of the human μ-opioid receptor in *Pichia pastoris*. *Protein Expr. Purif.* **2002**, 24(2), 212-220. https://doi.org/10.1006/prep.2001.1564.
5. Yurugi-Kobayashi, T.; Asada, H.; Shiroishi, M.; Shimamura, T.; Funamoto, S.; Katsuta, N.; Ito, K.; Sugawara, T.; Tokuda, N.; Tsujimoto, H.; Murata, T.; Nomura, N.; Haga, K.; Haga, T.; Iwata, S.; Kobayashi, T. Comparison of functional non-glycosylated GPCRs expression in *Pichia pastoris*. *Biochem. Biophys. Res. Commun.* **2009**, 380(2): 271-276. https://doi.org/10.1016/j.bbrc.2009.01.053.
6. Wang, X.; Sun, Y.; Shen, X.; Ke, F.; Zhao, H.; Liu, Y.; Xu, L.; Yan, Y. Intracellular expression of *Vitreoscilla* hemoglobin improves production of *Yarrowia lipolytica* lipase LIP2 in a recombinant *Pichia pastoris*. *Enzyme Microb. Tech.* **2012**, 50, (1), 22-28. https://doi.org/10.1016/j.enzmictec.2011.09.003.
